# A Spectrum of Pleiotropic Consequences in Development Due to Changes in a Regulatory Pathway

**DOI:** 10.1371/journal.pone.0043413

**Published:** 2012-08-24

**Authors:** Ana E. Escalante, Sumiko Inouye, Michael Travisano

**Affiliations:** 1 Department of Ecology, Evolution and Behavior, University of Minnesota, St. Paul, Minnesota, United States of America; 2 Departamento de Ecología de la Biodiversidad, Instituto de Ecología, Universidad Nacional Autónoma de México, Mexico City, México; 3 Biotechnology Institute, University of Minnesota, St. Paul, Minnesota, United States of America; 4 Department of Biochemistry, Robert Wood Johnson Medical School, Piscataway, New Jersey, United States of America; Fred Hutchinson Cancer Research Center, United States of America

## Abstract

Regulatory evolution has frequently been proposed as the primary mechanism driving morphological evolution. This is because regulatory changes may be less likely to cause deleterious pleiotropic effects than changes in protein structure, and consequently have a higher likelihood to be beneficial. We examined the potential for mutations in *trans* acting regulatory elements to drive phenotypic change, and the predictability of such change. We approach these questions by the study of the phenotypic scope and size of controlled alteration in the developmental network of the bacterium *Myxococcus xanthus*. We perturbed the expression of a key regulatory gene (*fruA*) by constructing independent in-frame deletions of four *trans* acting regulatory loci that modify its expression. While mutants retained developmental capability, the deletions caused changes in the expression of *fruA* and a dramatic shortening of time required for completion of development. We found phenotypic changes in the majority of traits measured, indicating pleiotropic effects of changes in regulation. The magnitude of the change for different traits was variable but the extent of differences between the mutants and parental type were consistent with changes in *fruA* expression. We conclude that changes in the expression of essential regulatory regions of developmental networks may simultaneously lead to modest as well as dramatic morphological changes upon which selection may subsequently act.

## Introduction

Heritable phenotypic change is a prerequisite for adaptive evolution. However, the process by which diversity in form originates, and the mechanisms that link phenotypic variation with genetic modification, are poorly understood. This is in part because the focus of traditional evolutionary theory has been on changes in gene frequencies and has not taken into account the complexity of biological systems that result in what we define as phenotypes (for a conceptual review see [1], [2]). New approaches have emerged to fill this gap, combining knowledge from molecular biology and evolutionary theory in the search for mechanistic information about the origin and evolution of phenotypic traits [3]–[5]. In this respect, the study of the evolution of development has become paradigmatic [2], and progress is being made in understanding the evolution of phenotype by comparative genomic studies and experimental manipulation of biological laboratory models [5]–[7].

The foremost example of the integration of molecular biology and evolutionary theory in the study of phenotypic adaptation is the evolution of developmental networks [8]. A commonly stated hypothesis is that the evolution of *cis*-regulatory elements in developmental networks, is less likely to cause negative pleiotropic consequences in the phenotype than *trans-*acting regulatory factors [7]. *Cis*-regulatory elements are closely linked to the loci that they affect, while *trans* regulators are either unlinked or distantly linked to the loci under their regulatory control. Because mutations in *cis*-regulatory elements impact closely linked loci, their effects are localized spatially or temporally, in contrast to mutations in *trans-*acting factors or structural genes that may affect global gene function [3]. The distinction among different mechanisms of regulatory control and structural genes has lead to the 'toolkit gene' concept [7], in which the localized expression of 'toolkit genes' can readily evolve via regulatory mutations. The prevalence of *cis*-acting regulatory elements within genomes provides a potential mechanism for decreased deleterious pleiotropy during evolution occurring via changes in development [7], which has been mainly evidenced by comparative genomic and expression studies [7], [9]. Within this framework, it is expected that inframe deletions of *trans*-regulatory elements within a developmental network will have pleiotropic effects resulting in substantial phenotypic change. However, the importance of *trans*-regulation remains contentious as the potential for localized expression appears limited. In this paper, we explore the scale of changes in developmental time and place, heterochrony and heterotopy, arising via *trans*-regulation, and determine if such phenotypic changes could facilitate adaptive evolution.

Another hypothesis for the importance of developmental networks in evolution is the potential for large beneficial effect mutations. The observation of mutations having dramatic morphological consequences, such as losing the ability for development of complex structures [10] or due to changes in developmental timing (e.g., [11]) suggests that large beneficial effects are possible. The importance of large effect mutations has a long and contentious history in evolutionary biology [12]–[14]. Nevertheless, mechanisms promoting abrupt phenotypic evolution remain a topic of intense interest [15]–[17], in particular the structuring of developmental networks into modules. A module is a highly interconnected sub-developmental network that has relatively few connections with other modules. This hierarchical network structure potentially limits the pleiotropic consequences of mutations across an entire developmental network, facilitating large effects beneficial mutations and limiting their deleterious effects. However, the debate on the adaptive potential of alterations in development persists, in part, because it is difficult to integrate genomic and phenotypic information [18], [19], and this is particularly true for complex traits. The lack of clarity on the mechanisms by which development affects evolutionary outcomes, undermine its utility in a reformulation of evolutionary theory [20].

We have initiated a research program to directly investigate developmental evolution using a model system that is both relatively simple to propagate and is genetically tractable, the free-living microbe *Myxococcus xanthus*. *M. xanthus* undergoes multicellular development as a social behavior via aggregation of vegetative cells and formation of fruiting bodies [21]. It is readily culturable in laboratory settings [22] and can be genetically manipulated with relative ease, allowing us to directly observe the developmental consequences of specific genetic changes [23]. Development in *M. xanthus* occurs when resources become scarce and individual cells migrate towards aggregation centers, gliding to form multicellular groups consisting of about 100,000 cells. These groups of cells (swarms) develop into fruiting bodies (FBs), each containing approximately 10,000 spores, after 24–72 hours via a series of temporally and spatially structured cellular activities [24], [25]. Phenotypic variation resulting from variation in social traits (such as aggregation to form FBs), has been reported as prevailing in natural microbial populations [26]–[29] suggesting the value of such variation in competition and its potential for adaptation.

**Figure 1:**
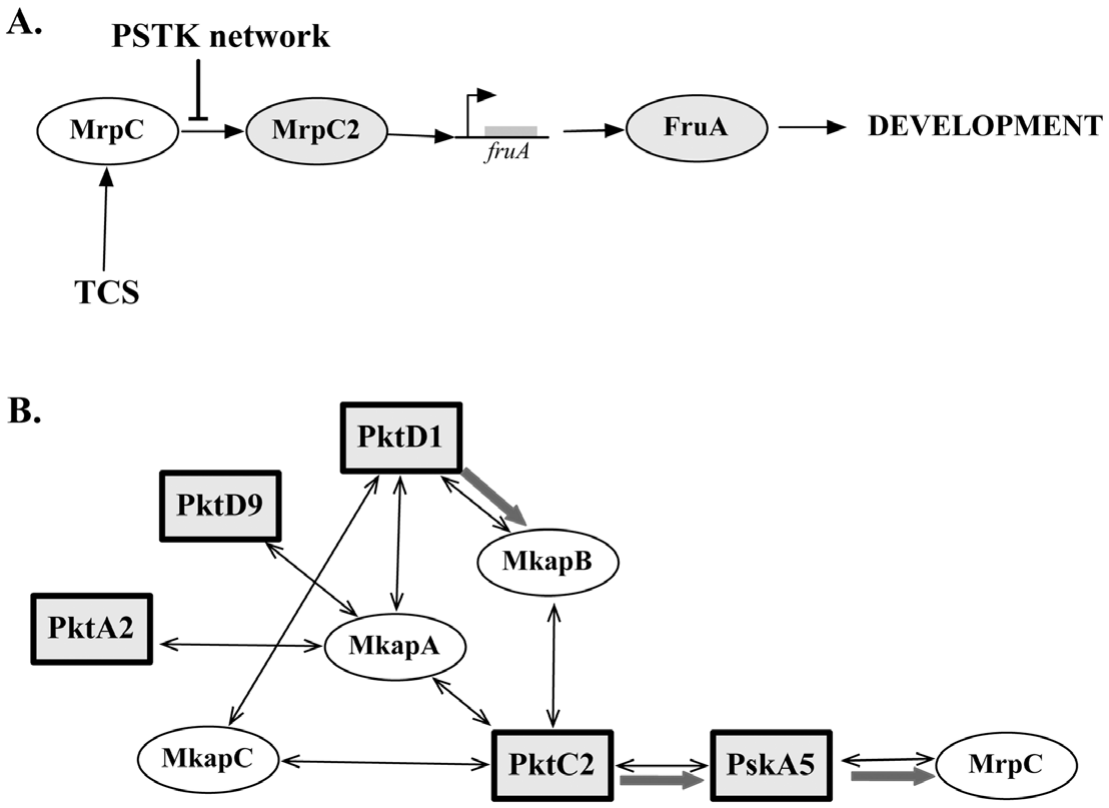
Regulatory network model of development in *Myxococcus xanthus*. A. Regulatory network model of *fruA* expression. Expression of *fruA* is a key step in the induction of developmental gene expression and the achievement of multicellular fruiting body formation and sporulation. During vegetative growth, *mrpC* is transcribed at low levels, using its own product (MrpC) as a transcription factor (positive feedback). When starvation signals trigger the expression of genes in a two component system (TCS) the newly synthesized MrpC is not phosphorylated, but is instead processed to MrpC2, which has a higher affinity for the *mrpC* and *fruA* promoter regions [34]. A PSTK network inhibits development by phosphorylating MrpC. B. The Protein Serine-Threonine Kinase (PSTK) network model. PSTK network is thought to consist of at least five kinases (squares) as well as three multikinase associated proteins (Mkaps). Double-headed arrows indicate interactions identified by yeast-two hybrid screens, while gray arrows are characterized phosphorylation pathways [35]. PskA5, a protein kinase activated by PktC2, phosphorylates MrpC, reducing its affinity for both *mrpC* and *fruA* promoter regions, and preventing untimely initiation of development. In this paper, PktA2, C2, D1 and D9 were deletion targets. The *pskA5* locus is closely linked to that of *mrpC*/*mrpC2* and therefore was not a candidate for in-frame deletion.

In this study we investigate the phenotypic consequences of regulatory changes in development, focusing on changes in developmental timing and outcome. What are the phenotypic consequences of mutations in *trans-*acting regulatory pathways? The pathway of signal dependence through *M. xanthus* development is an area of active research, but several key steps have been identified that involve both intra- and extracellular signaling [21], [25]. One essential step is appropriate expression of the *fruA* gene, a key intracellular regulator of development that is required for cell aggregation and fruiting body maturation [30]. Regulation of *fruA* expression occurs via multiple signaling pathways that have been partially elucidated (Figure 1). We constructed in-frame deletions of four regulatory loci whose gene products have been tentatively identified as affecting *fruA* expression and fruiting body development [31]. Loci were chosen based on previous data indicating that their loss did not prevent development in *Myxococcus xanthus* [31]–[34], the importance of serine-threonine phosphate cascades in development [35], and their association with the *fruA* regulatory network. In-frame deletions were used as they have unambiguous effects on expression of a regulatory locus, and they have no or little effect on expression of upstream or downstream loci linked to the deleted gene. Although information exists on the pleiotropic effects of regulatory mutations in *M. xanthus* model [35], there have not been studies interpreting such observations within an evolutionary framework. We designed assays to assess phenotypic change due to regulatory mutations relative to unmutated genotype and unexpected variance. This design provides a straightforward approach to compare traits and the scale of mutational effects. Thus, the present experimental and analytical approaches on the impact of regulatory mutations provides 1) direct information on their phenotypic effects that is otherwise difficult or impossible to achieve by other approaches such as comparative genomics and 2) evidence emphasizing the utility of a simple biological model in the study of phenotypic evolution.

The in-frame deletions caused dramatic shortening of time required for fruiting body development, consistent with the anticipated effects of loss of the four regulatory loci. We found phenotypic changes in the majority of traits measured, indicating pleiotropic effects of changes in regulation. The magnitude of the change for different traits was variable but the extent of phenotypic differences among the mutants and parental type were consistent with linear changes in *fruA* expression. These results show that multiple phenotypic changes in developmental traits can readily occur due to pleiotropy, via simple genetic changes affecting development in a predictable fashion [36].

## Results

### Changes in *fruA* Expression

**Figure 2:**
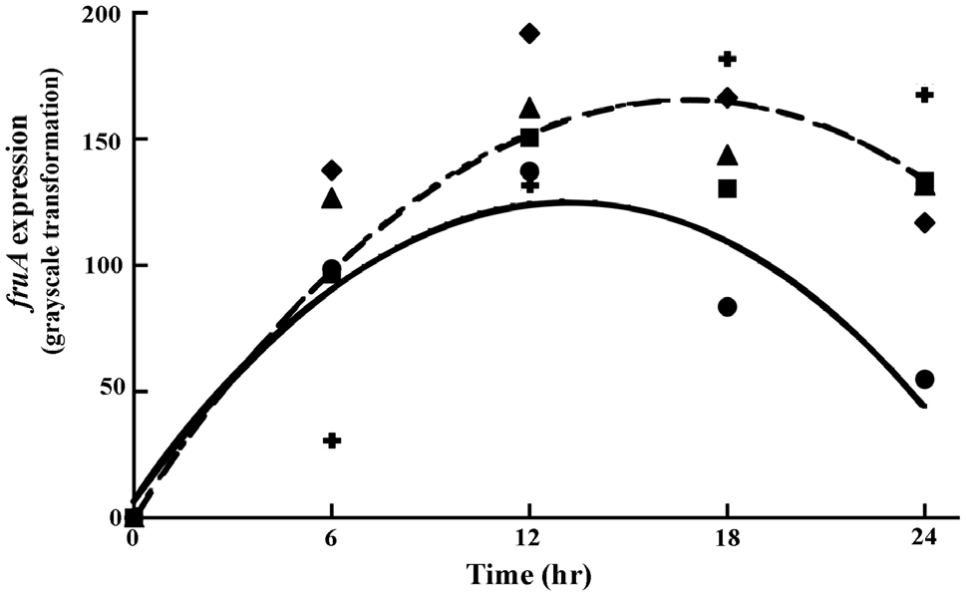
Expression of *fruA* over time for all strains. Code for strains are: DZF1 (circle), A2 (square), C2 (cross), D1 (triangle), and D9 (diamond). Solid and dashed lines correspond to the parental and knock-out mutant strains respectively.

Prior studies suggested that deletion of the *pktA2*, *pktC2, pktD1* and *pktD9* loci would alter *fruA* expression and thereby impact development and fruiting body formation [31]–[34]. We observed large changes in *fruA* expression during development of the mutant knockout strains. The observed differences in expression, between the knockouts and the parental strain, are consistent with the predicted regulatory structure of *fruA* (Figure 1). In particular, the mutants have an overall higher level of expression (F_1,10_ = 9.8, *p* = 0.0107, ANCOVA adj. r^2^ = 0.875), as determined by a planned contrast of mutants versus parental strain. Temporal expression of *fruA* differs between the mutants and parental strain, as assessed by the interaction of genotypic state, mutant or parental, versus time (F_1,4_ = 9.62, *p* = 0.0362,, ANCOVA adj. r^2^ = 0.915). After 12 hours into development, the average expression of *fruA* for the mutants, is higher than the parental stain, and it is also maintained for longer time (Figure 2).

### Phenotypic Traits

To determine the size and scope of phenotypic consequences of the developmental regulatory changes, we measured the rate of developmental progression, several fruiting body characteristics including size, variance in size, number, as well as spore number and viability. Exponential growth rate was our measure of the vegetative phenotype.

**Figure 3:**
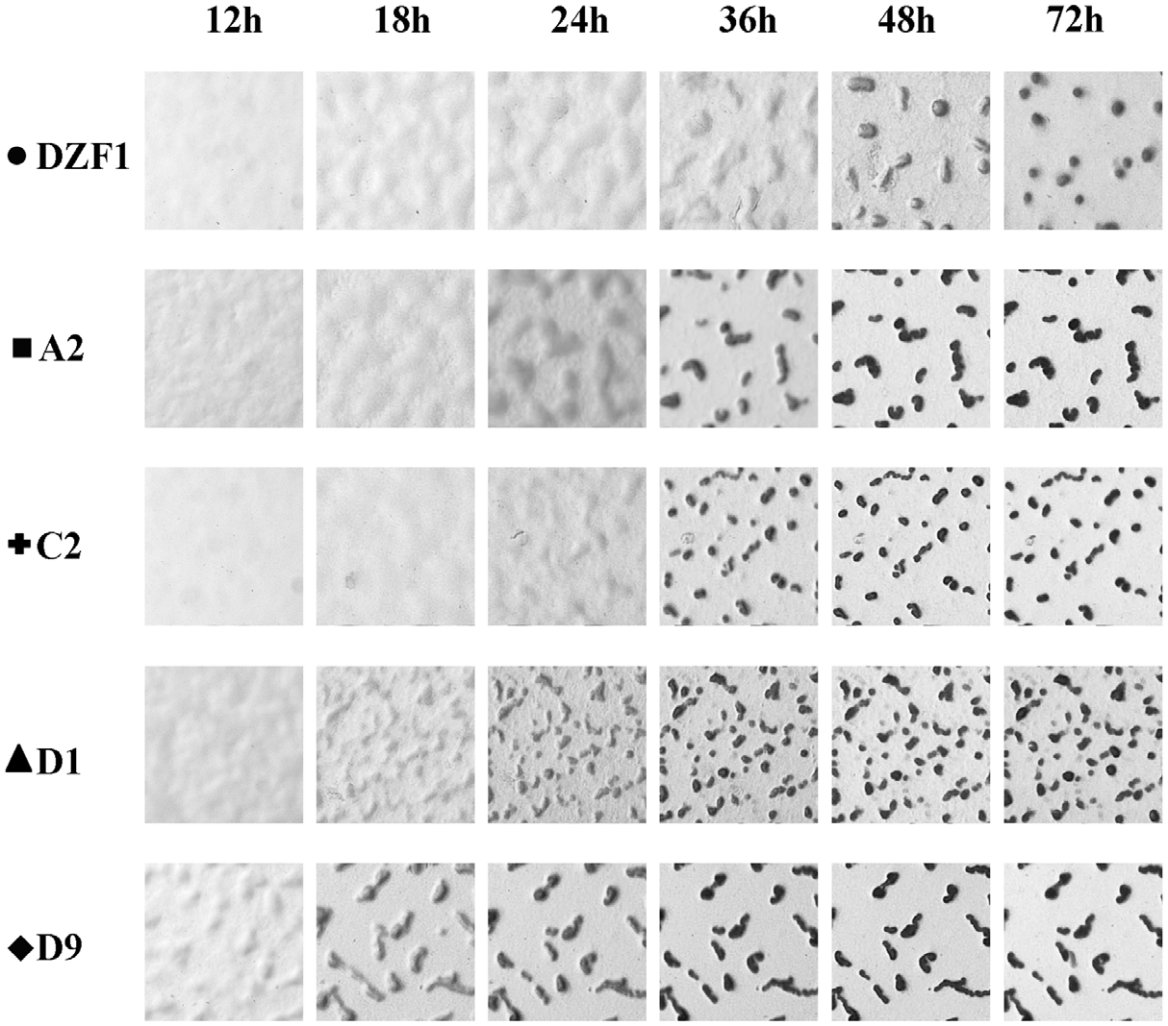
*Myxococcus xanthus* parental strain and knock-out mutants fruiting body formation at 12, 18, 24, 36, 48 and 72 h development. Micrographs were taken at 269.5 pixel/mm on TPM plates. Different mutations account for changes in developmental timing, which also has consequences in the final shape and distribution of fruiting bodies.

**Figure 4:**
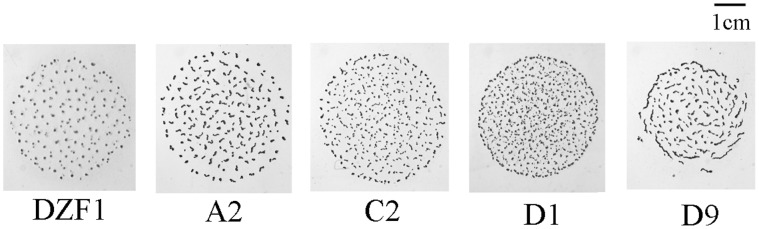
Phenotypic diversity after 72 h development of *Myxococcus xanthus* parental strain and knock-out mutants. Solid dark spots correspond to mature fruiting bodies containing myxospores after aggregation and differentiation of vegetative cells. Observed diversity results from knocking out genes associated with changes in developmental timing. Micrographs were taken at 269.5 pixel/mm on TPM plates.

**Figure 5:**
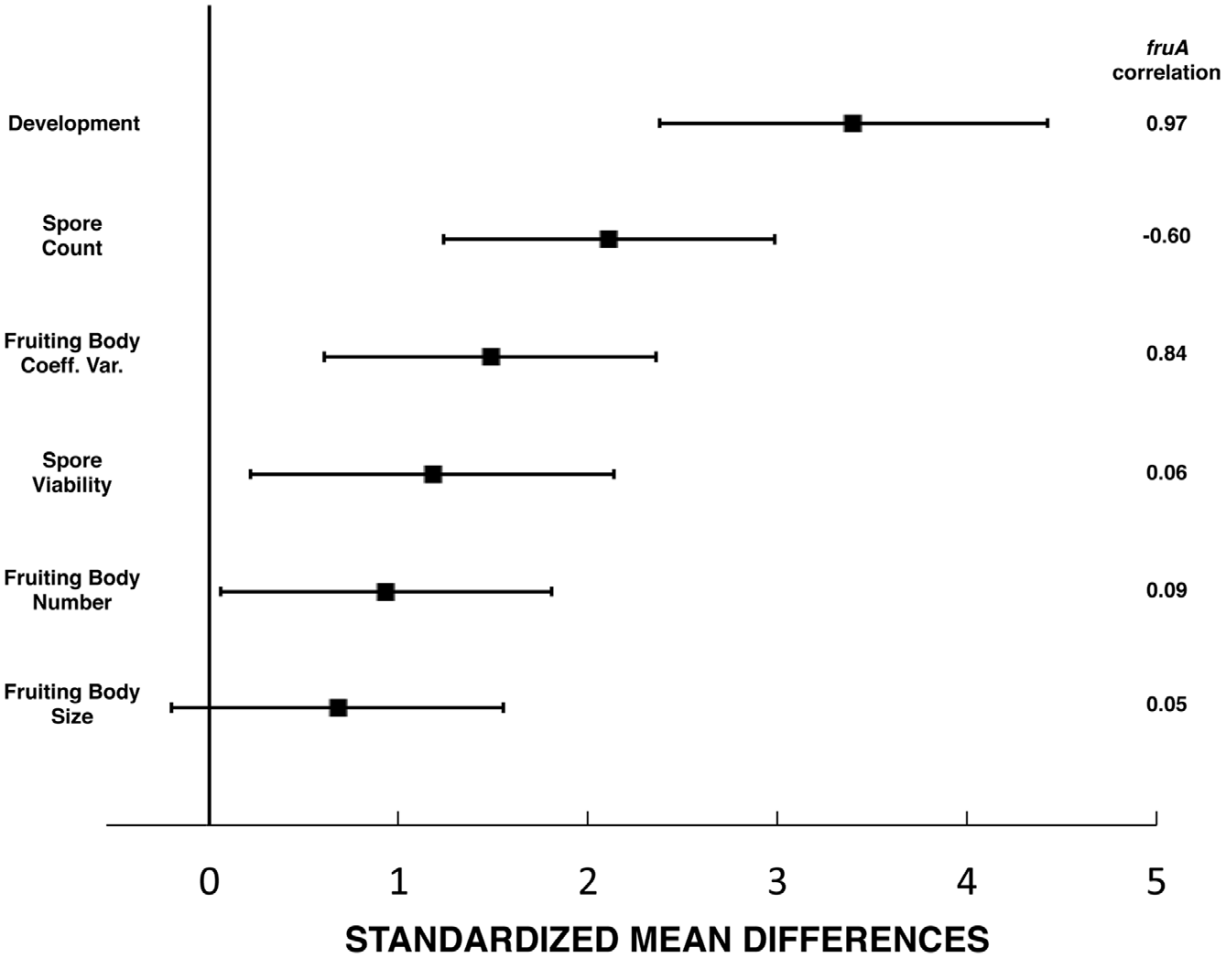
Standardized mean difference among mutants and parental strains. Error bars are 95% confidence intervals (CI) for the observed phenotypic variation. Statistical differences between parental strain and mutants are supported by exclusion of ‘0′ within the confidence intervals. The right-hand column shows correlation coefficients for differences among the mutant strains with *fruA* expression.

Parental and mutant strain development differs greatly (Table 1, Figures 3 and 4). Development, measured as progression rate into mature fruiting bodies, is accelerated in all mutants relative to the parental genotype DZF1. The largest amount of genetically determined phenotypic variation among the five genotypes is observed at 36 hours (analysis not shown), at which point all mutants have completed or nearly completed fruiting body development. Development is complete for all the mutants after 48 hours, while it is substantially slower for the parental genotype (*p* = 2.2×10^−6^) by an average of 19.2%. Even so, not all fruiting body size traits are affected by the in-frame regulatory deletions, and there were large differences in the phenotypic consequences depending upon the trait (Table 1, Figure 5). For example, fruiting body mean size is not significantly different between the mutants and DZF1, but variance in size is far larger in the mutant strains compared with DZF1, which is notably homogeneous in the size of the mature fruiting bodies. Significant differences in the number and viability of resulting spores are observed between DZF1 and mutants, some mutants produce more spores than the parental strain and in all cases relative viability is diminished in the mutants. In this study we focus on the potential for phenotypic changes to occur, and spore production and viability are only considered as phenotypic traits that are assessed for change (rather than measures of fitness).

**Table 1:** Variation among genotypes for developmental traits.

	Genotype^a^				Replicate^a^				Block^a^				Replicate x Block^b^				Error	
Trait	MS	Df	F	p	MS	df	F	p	MS	df	F	p	MS	df	F	p	MS	df
Development^c^	0.0696	4	22.5	0.0004	0.0245	2	2.07	0.205	0.002	3	0.271	0.845	0.012	6	3.92	0.049	0.0031	7
FB CV	3184	4	3.61	0.318	2950	2	3.35	0.065	71.87	3	0.816	0.969	–	–	–	(0.99)	881	14
FB number^d^	0.064	4	3.57	0.033	0.026	2	1.43	0.271	0.027	3	1.51	0.255	–	–	–	(0.46)	0.18	14
FB size	20271	4	1.56	0.24	42523	2	3.28	0.068	17113	3	1.32	0.31	–	–	–	(0.15)	12961	14
Spore Count^d^	0.318	4	7.44	0.002	0.031	2	0.735	0.497	0.074	3	1.74	0.205	–	–	–	(0.22)	0.598	14
Spore Viability^d^	0.338	4	2.67	0.084	3.54	2	27.97	3×10^−5^	0.139	3	1.10	0.388	–	–	–	(0.95)	0.126	12

a Random factor.

b Partial F-test values in parenthesis for inclusion of an interaction term in the analyses. In only one instance, for development, did a partial F-test indicate that including the interaction term statistically improved the analysis.

c Assessed at 36 hours.

d Analysis carried out on Log_10_ transformed data.

Far fewer differences among mutant strains were observed for phenotypic traits (Table 2). Statistically significant differences among mutants were observed for development (F_3,7_ = 10.92, p = 0.005), with mutant genotype D9 proceeding through development faster than A2. Mutants C2 nor D1 were indistinguishable from one another or A2 and D9. No other statistically significant differences were observed (analysis not shown).

**Table 2:** Mutant trait means and 95% Confidence Intervals (CI).

	Genotype			
Trait	A2	C2	D1	D9
Development^a^	0.68±0.42^b^	0.82±0.1	0.74±0.3	0.92±0.22
FB CV	107.78±148.21	98.04±113.73	93.46±40.66	126.53±120.69
FB number^c^	2.36±0.40	2.49±0.23	2.57±0.43	2.37±0.54
FB Size	413.92±377.61	295.99±245.46	342.09±471.50	364.14±193.24
Spore Count^c^	6.22±0.30	6.02±0.36	5.94±0.23	6.09±0.51
Spore Viability^c^	5.08±0.90	4.77±1.36	4.18±1.03	4.77±2.17

a Assessed at 36 hours.

b 95% Confidence intervals determined by a *t*-distribution with n −1 = 2 df.

c Analysis carried out on Log_10_ transformed data.

### Statistical Measurement of Scope and Size of Phenotypic Consequences

To simultaneously compare phenotypic effects across traits, we scaled each measure to their respective standard deviations [37]. This allowed us to determine the phenotypic variation that was attributable to differences between DZF1 and mutants and to gauge the size of the differences in the same units. This approach provides values that are in units of standard deviation (Table 3), so that comparable 95% confidence intervals can be generated (Figure 5). Confidence intervals excluding zero indicate statistically significant differences between the parental genotype (DZF1) and the mutants, not corrected for carrying out multiple simultaneous tests. Confidence intervals excluding 1 indicate the differences between DZF1 and mutants are greater than the non-genetic component of the phenotypic variation (Figure 5). The results show three things. First, the size of the change for different traits was variable and most traits measured were affected by the regulatory change in development. Second, 5 out of 6 traits have statistically significant phenotypic differences when comparing mutants with parental strain (DZF1). Finally, 3 out of 6 trait differences remain statistically significant even after carrying out sequential Bonferroni correction for multiple tests.

**Table 3:** Differences between the parental and mutant genotypes for developmental traits.

Trait	Mean Difference	t_S_	df	p	SD_pooled_	Standardized Mean Difference
Development	0.189	7.841	7	0.0001^a^	0.056	3.399
FB CV	44.01	3.632	14	0.0027^a^	29.68	1.483
FB number	0.124	2.281	14	0.039	0.134	0.931
FB size	77.15	1.66	14	0.119	113.8	0.678
Spore Count	0.436	5.17	14	0.0001^a^	0.207	2.109
Spore Viability	0.419	2.67	12	0.020	0.356	1.18

a Statistically significant after sequential Bonferroni correction for carrying out multiple simultaneous tests.

We evaluated the correlation of phenotypic changes and gene (*fruA*) expression changes (Figure 5, right hand column), noting a relationship between differences in phenotype between the mutant and parental strains with *fruA* expression. The differences among trait responses is consistent with differences in *fruA* expression, as supported by a linear regression of SMD on the square root of the of the absolute correlation values for *fruA* expression and each phenotypic trait (slope = 2.26, t_4_ = 2.81, p = 0.048, adj. r^2^ = 0.58).

**Figure 6:**
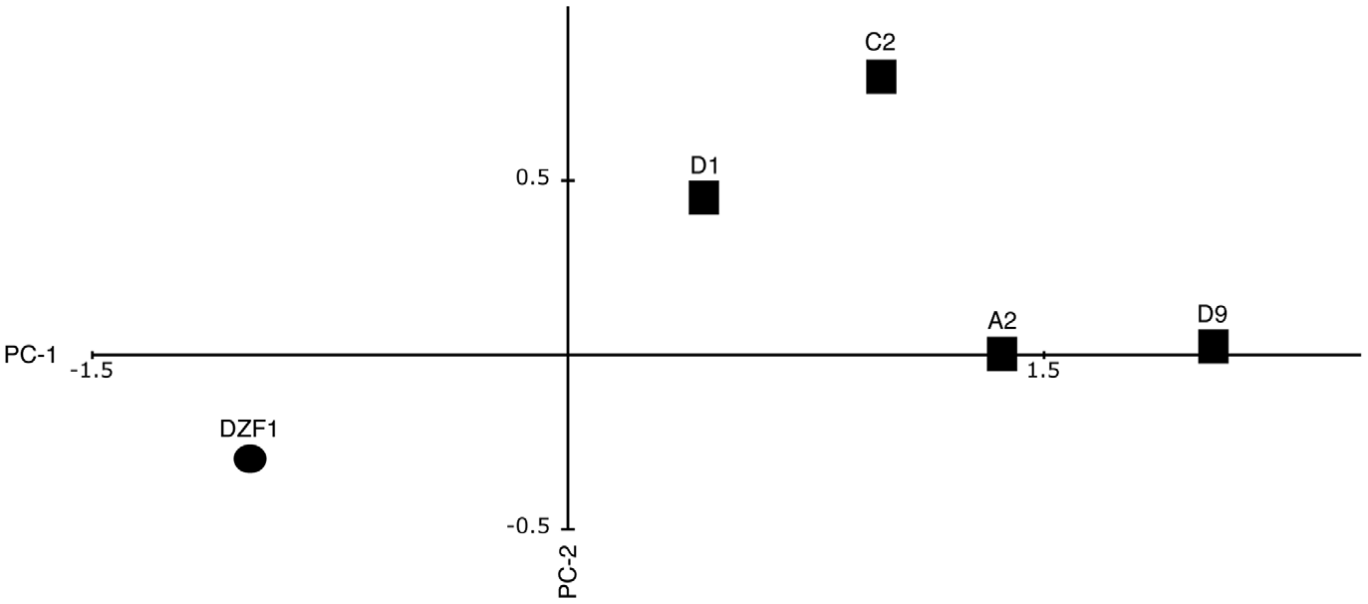
Principal Component Analysis of all strains. Traits included in the analysis are: log_10_ terminal fruiting body count, log_10_ terminal viability Count, log_10_ terminal spore count, 36 hour development, coefficient of variation for fruiting body size at 72 hours, and fruiting body size at 72 hours, PC-1 and PC-2 account for 40% and 28.3% of the phenotypic variance, respectively.

We also performed a principal components analysis of developmental traits, to assess the size and scope of statistically independent traits. While conclusions from the above analyses of individual traits are potentially limited, since the data for different traits were collected from same replicates and are therefore not independent, the structure of data collection allows for a simultaneous analysis of the developmental traits via a principal component analysis. Three components were statistically significant by a chi-square test (p<10^−5^, 10^−4^, and 10^−2^, respectively), accounting for total of 86.7% of the variation (40.0, 28.3, and 18.3, respectively). An ANOVA on the composite principal component trait values indicates that the genotypes are readily distinguished (Figure 6) for the first (F_4,11_ = 6.78, p = 0.0053) and second axes (F_4,11_ = 4.40, p = 0.023), but not the third (F_4,11_ = 1.68, p = 0.224). No statistically significant differences were detected among the mutants when considered alone, without the unaltered parental genotype. More importantly, the parental and mutant genotypes are statistically distinct, as determined by t-tests on the primary (t_11_ = 5.195, p = 0.0003) and secondary (t_11_ = 3.295, p = 0.007) axes. Their respective SMD are 1.92 and 1.23.

### Growth Rate

A statistically significant difference between the parental genotype and the knockout mutants was observed (F_4,8_ = 6.81, *p* = 0.011), due to the decreased growth rate of one mutant (D1). No differences among genotypes for growth rate were observed when D1 was excluded from the analysis (F_3,6_ = 1.9, p>0.2), and there was no evidence that the growth rate of parental genotype differed from the other three knockout mutants (F_1,8_ = 0.81, p>0.4). The decreased growth rate of the D1 genotype indicates that there is ‘crosstalk’ by at least some regulatory elements to affect both development and vegetative growth.

## Discussion

A major success of the Modern Synthesis was the abstraction of genetics. By focusing on the intersection of Mendelian genetics and Darwinian selection, a general evolutionary theory was developed. Nevertheless the limitations of the purely genetic approaches have long been apparent [38], [39], as they largely ignore the complexity of biological systems and fail to incorporate mechanistic details underlying phenotypic differences on evolution [40], [41]. In the absence of mechanistic information, the evolutionary intricacies underlying complex phenotypes remain unclear.

This study is one step towards a functional synthesis [42] on the evolution of development. The approach taken combines the rigor of experimental molecular biology with the conceptual foundations provided by evolutionary biology (see also [43]). In this study, we investigated the potential for adaptation by developmental modification. We were interested in *trans*-regulators of development, as they seem to be critical for development, and there is relatively little quantitative information to determine if changes in *trans*-regulation could give rise to the kind of phenotypic changes that would allow evolution to proceed. Our approach involved the investigation of scope and size of phenotypic consequences by alteration of a developmental network. This topic is relevant since it remains unclear how changes in developmental timing alter phenotype and adaptation as a consequence.

One model of developmental evolution states that morphological changes are more likely to occur through changes in the expression of “toolkit” loci via their promoter regions (*cis-*regulation). These “toolkit” loci encode functionally conserved proteins of mosaically pleiotropic influence within the vast regulatory network they control. Structural changes in them are presumed to be less tolerated because their large deleterious consequences in fitness [7]. Despite debate on the molecular nature of morphological change, it is clear that expression changes in these toolkit genes do occur and are associated with morphological modifications in animals. There is substantial evidence of their functional and sequence conservation across phylogenetic groups, like the Hox family of transcription factors. Nevertheless, the use of animal models in evolutionary development studies imposes inherent practical complications, as well as potentially limited interpretation. Moreover, these studies have primarily focused on among species comparisons, unlike the within species differences examined in this study.

In the *Myxococcus* model, the importance of toolkit genes in the evolution of development is unclear, as many developmentally essential genes are not conserved across different Myxococcales species [44]. We perturbed the expression of an essential gene (*fruA*) by constructing independent in-frame deletions. The deleted loci were previously identified as associated with the *M. xanthus* developmental network, and were hypothesized to impede the onset of development by a phosphate cascade terminating in transcriptional regulation of *fruA* gene expression [31]–[34]. By generating precise in-frame deletions, we observed changes in developmental timing and altered *fruA* expression, verifying expectations. Moreover, the extent of change in other developmental traits was consistent with altered *fruA* expression, suggesting, that large changes in the timing of developmental networks can occur by proportional changes in the underlying mechanisms by which they occur. The observation that phenotypic variation is linearly associated with changes in gene expression of an essential gene shows the potential for diversification by simple mutations in *trans*-regulatory elements of a developmental network. Predictable, and potentially gradual, evolutionary change (see [36]) in developmental traits can proceed by changes in *trans* regulation without catastrophic consequences.

We devised planned comparisons between mutated and unmutated strains to specifically assess phenotypic change due to regulatory mutations, relative to the unexplained (error) variance in phenotype. This experimental design provides a straightforward approach to compare not only traits but to scale the effects of mutations.

### Scope of Phenotypic Consequences

Statistically significant differences in five of six developmental traits, three after Bonferroni correction, were observed. Phenotypic consequences were evident across a range of traits, as seen by differences in spore count and fruiting body size variation within a developmental swarm. Moreover, substantial phenotypic effects were observed for the statistically independent first and second principal component axes, strongly supporting pleiotropic consequences. The observation of complex modes of developmental evolution is not restricted to our study, as discussed in a recent reevaluation of the Hox gene evolution [45]. A study by Liao and colleagues [5] suggests that genes associated with anatomical or morphological changes are in general more pleiotropic than genes involved with physiology. The results are part of a comparative genomics study predicting that morphological evolution more often should involve transcriptional regulation and gene expression changes. However, interpretation of these and results from other complex systems, such as human and mice, are necessarily difficult, in that assigning gene function and mode of activity is rarely unambiguous.

### Size of Phenotypic Consequences

We measured the size of phenotypic changes by statistical analysis of morphological effects of mutations affecting developmental timing. We observed a gradient in the magnitude of consequences ranging from 0.68 to 3.4 standard deviations of within-genotype phenotypic variation. The large reduction in developmental timing illustrates the potential for dramatic alterations in developmental programs, without catastrophic consequences. The extent of phenotypic change in a trait was largely consistent with patterns of *fruA* gene expression. It is particularly interesting to note that one of the traits that differs between mutants and the parental strain is FB size variation (CV), and we hypothesize that this could be the result of heterotopy. Fruiting body formation in *Myxoccocus* is affected by cell-cell communication and extracellular signals [46], that involve population density and nutrient availability [25]. Alteration of *fruA* gene expression changes the timing of development and thus may alter the consequences of extracellular signals. In our experimental system, heterochronic differences in expression of development-related genes across mutants lead to differences in fruiting body size. As stated by Carroll [7] “*changes in the spatial regulation of toolkit genes and the genes they regulate are associated with morphological divergence*”. We hypothesize that heterochronic differences in *fruA* expression resulted in heterotopic differences in fruiting body size due to gradients in extracellular signals. These observations suggest that evolutionary change of phenotypic traits could be either gradual or discrete, depending on the traits, loci and mutations involved.

In addition to the planned comparison between mutants and unmutated parental genotype, variation among mutants may exist. For instance, mutant D9 appears to have faster development (Figure 3), larger fruiting bodies (Figure 4), and the most variance in FB size (Figure 3). Despite these observations, statistical significance for trait differences among mutants was only observed for speed of development, between mutants D9 and A2. Developmental traits are highly environmental labile, and the primary focus of the study was contrasting the parental and mutant genotypes. Moving forward, it would be worthwhile to investigate the apparent differences among mutants that were not predicted, using our current results to frame expectations. These subsequent experiments would necessarily need to be carefully executed, and blocked accordingly, as our current three-fold measurements are insufficient to discriminate the mutant phenotypes with statistical rigor.

### Genetic Basis for the Change in Form

A main goal in the study of morphology is to determine the underlying mechanistic bases for its evolution. Our observations suggest that there is a direct correlation of morphological changes of developmental phenotypes with changes in the expression of a key gene for development in *M. xanthus*. We also observe that there are differences in this correlation depending on the relative position of the gene affected in the network, as is the case for A1, D1, D9 compared to C2. These differences were not anticipated, despite differences in the hypothesized regulatory pathway (Figure 1, [35]). We anticipated similar roles of the four regulatory loci, with differences arising via magnitude of the effects. Future work disentangling the differences between these two groups will be helpful in further understanding the consequences of perturbing different parts of the regulatory network. Finally, we observed that the extent of phenotypic differences among the mutants and parental type are largely consistent with *linear* changes in *fruA* expression, indicating the major changes in the timing of developmental networks can occur by proportional changes in the underlying mechanism by which they occur. The finding of phenotypic variation correlated with changes in genetic expression, shows the potential for diversification by simple mutations in the regulatory network. Mutations altering social behavior and development potentially lead to evolutionary change in phenotype without catastrophic consequences. These observations may help future studies in looking for links between development evolution and natural patterns of phenotypic variation, such as those observed in populations of *M. xanthus* by Kraemer and colleagues [29].

### Evolutionary Implications of Variation in Developmental Timing

Although most studies of developmental process in *M. xanthus* have been conducted with laboratory strains in controlled laboratory settings, there is documented evidence of the globally widespread presence of developmentally competent strains [47]. This widespread behavior is nonetheless rapidly lost if selection for social behavior is relaxed [22], [48], indicating that social proficiency and development into FBs is highly beneficial in the wild. Moreover, work by Kraemer et al. [29], demonstrate natural variation in developmental timing of strains recovered from different sites, suggesting that variation in selective forces across different environments may contribute to the persistence of such variants. At this point is impossible to know if such forces act directly on developmental timing or in other pleiotropically linked trait(s) (such as social motility), but speculation on the selective advantage of developmental timing can be made. For example, if two different populations of strains are mixed, one being faster in developing than the other, it is possible to imagine that the faster developer might monopolize signaling molecules and exclude the slower developer from producing viable spores [29], this being a plausible mechanism for genetic differentiation and, potentially, speciation. If the trait under selection is, for example, social motility, which is linked with predatory efficiency and development, it is easy to imagine that slow-developing strains can have an advantage when resources are scarce, by using them for longer before going into development. This being said, the frequency with which *M. xanthus* goes into development in the wild is completely unknown, as is the contribution of such behavior to overall fitness or adaptation [49].

### Conclusions

This study provides evidence that emphasize the utility of a simple microbial model for research on developmental evolution and the consequences of phenotypic diversity generation. This is of particular relevance because of the dependence on gene-phenotype mapping in the search for understanding the mechanisms underlying the origin and evolution of complex traits. The *Myxococcus xanthus* model for development used here provided detailed quantitative measurements of phenotypic consequences resulting from changes in the regulation of development. We observed that simple genetic perturbations of the signal cascade for development result in significant pleiotropic changes in phenotype that range in magnitude. Our results imply that changes in *trans* acting regulatory regions can potentially lead to predictable phenotypic evolution.

## Materials and Methods

### Strains and Mutant Construction

The strains used included the parental strain *Myxococcus xanthus* DZF1 [50] and 4 single PSTKs (Protein Serine/Threonine Kinases) in-frame deletion mutant strains. Mutants were constructed using the kanamycin resistant gene (kan) for positive screening and a galactokinase gene (galK) for negative screening. Briefly, two DNA fragments of approximately 600 bp in size were amplified by PCR using the genomic DNA as a template. Fragment 1 contained the 600-bp upstream region of the translation initiation codon with the first several amino acid codons and fragment 2 was the 600-bp downstream of the translation termination codon with several amino acid codons, also a unique six-base cutter restriction enzyme site was introduced upstream, and downstream the target sequence. This permitted construction of inframe-deletion mutants. Fragments 1 and 2 were cloned into pKO1kmr carrying the galK and kan genes. After the constructed plasmid was introduced into wild-type cells, the plasmid with the wild-type gene was eliminated by the addition of D-galactose in a medium [23]. The strains used were: Parental strain (DZF1), *ΔpktA2* (A2-1), *ΔpktD1* (D1-4), *ΔpktC2* (C2-2), and *ΔpktD9* (D9-2). Multiple vials for each isolate were frozen (20% glycerol) and stored at −80°C until used. None of the four deleted loci *pktA2* (MXAN 1467), *pktD1* (MXAN 4017), *pktC2* (MXAN 1710), and *pktD9* (MXAN 6420) are adjacent to one another or to the *mrpC/mrpC2* (MXAN 5125) locus. The *pskA5* locus is nearby to the *mrpC/mrpC2* locus. MXAN number designations refer to the sequence annotations of the *M. xanthus* genome [50].

### Microbiological Procedures

To revive strains from the frozen storage, stocks of each strain were thawed and 50 µl spotted into a CYE plate (1% Bacto Casitone, 10 mM Tris-HCl (pH 7.6), 0.5% yeast extract, 10 mM MOPS (pH 7.6) and 4 mM MgSO_4_) [35]. Inoculated plates were incubated at 30°C for 3 days. After this, cells were picked with a loop and used for further experimentation.

All assays for vegetative phenotype were performed using CYE plates or broth and for developmental phenotype TPM plates or solution were always used: 10 mM Tris-HCl (pH 7.6), 1 mM K_2_HPO_4_, 8 mM MgSO_4_ [43].

### Quantification of Pleiotropic Effects

All the phenotypic measures for each strain were performed in triplicate and in blocks to give statistical support to the observed phenotypic measurements and to rigorously evaluate potential pleiotropic consequences in the resulting phenotypes.

### Vegetative Phenotype

#### Growth rate

Growth on CYE broth is vegetative with no social predatory behavior or Fruiting Body (FB) development. For growth measurements, cultures were grown in 250-ml Erlenmeyer flasks with Klett tubes attached. All the inoculated flasks were incubated at 30°C with shaking (250 rpm) to keep the cultures well oxygenated. Growth was measured with a Klett-Summerson colorimeter [50], using amber filter (No. 66) with transmission 640 to 700 nm (Klett Mfg. Co., Inc).

### Developmental Phenotype

We quantified development both as for timing and for final phenotypic results. For timing we analyzed darkness of FBs as a measure for cell aggregation and FB maturity [43], and *fruA* expression at different time points. We also assessed FB number, FB size, FB size variation, total spore counts and viable spore counts.

#### Gene expression assay

Protein samples for Western blot analysis were prepared from cells developing on TPM plates. 10 µl of the cell suspension prepared as described above were spotted at 64 spots per a square plate (8 cm × 8 cm). The developing cells were harvested from 2 plates at the indicated time points, suspended in ice cold 500 µl TM buffer (10 mM Tris-HCl: pH 7.6), 8 mM Mg_2_SO_4_), and precipitated. The precipitated cells were kept at –80°C until used. The cells were solubilized in 100 µl sample loading buffer and heated for 5 min in boiling water with vigorous vortexing. Cell lysates were quantified using a Bradford assay (Bio-Rad Laboratories). Protein lysates (15 µg) were resolved by 12% SDS-PAGE and transferred to polyvinylidene difluoride (PVDF) membrane using semidry transfer apparatus (Bio-Rad Laboratories). Western blot analysis was performed using anti-FruA IgG, anti-CsgA (P17) IgG and anti-Tps polyclonal antibodies. Secondary goat anti-rabbit IgG-alkaline phosphatase (AP) conjugate (Bio-Rad Laboratories) was used according to the manufacture’s protocol.

#### Development assay

Strains were propagated in CYE broth at 30°C and 250 rpm until an approximate optical density of 100 Klett units was reached (4×10^8^ cells/ml). Cells were harvested at that moment by spinning them down using a microcentrifuge (6000 rpm × 10 min) and washing off remnant nutrients using TPM solution (10 mM Tris-HCl (pH 7.6), 1 mM K_2_HPO_4_, 8 mM MgSO_4_) [51]. After washing, the cell pellet for each strain was resuspended in 1/10th of the original volume. To evaluate developmental behavior of each strain, 15 µl of the cell suspension was spotted onto TPM plates (1.5% agar). The plates were prepared 2 days in advance to avoid excess moisture and pre-warmed for 20 min at 30°C before each cell suspension is spotted. The spots were dried for 20 min and then incubated at 30°C for 4 days. While incubating the plates, FB formation (cell aggregation) was assayed by taking stereomicroscope photographs at different time points (0, 12, 18, 24, 36, 48, and 72 hours) using a Nikon SMZ1500 Zoom Stereo Microscope. All the images were saved in a digital format, processed, and analyzed using ImageJ software [52].

#### Developmental phenotype measurements

- *fruA* expression. Digital images of the expression assay gels were processed to obtain quantitative measures. We used the Histogram Analysis tool implemented in Image J [52] and obtained the amount of ‘black’ in the image, as a direct correlate of the gene product (FruA). In this way, we were able to assess gene expression at different time points during development for the parental strain and mutants.
- Fruiting body developmental timing. We used an increase in coefficient of variation between pixels as a measure of fruiting body (FB) maturation, and the time sequence of images was used to estimate the FB timing. As FBs develop, they darken and we measured the change in color over time for each mutant. Color change was measured using the Histogram Analysis function implemented in Image J [52] and this tool makes it possible to measure the black/white distribution of pixels in the image. The two extremes of the distribution are: 0 h time point when cells are first spotted onto the plate and 72 h time point when all genotypes have completed development. When the cells were first spotted they cannot be distinguished from the background and are homogenously distributed. At 72 hours, mature FBs contrast strongly with the background, and the variance in the color (black and while) across pixels will be >0. To standardize the variance values, each time point variance value was divided by the final 72 h value, so all samples had a variance of 1 at the time point 72 h. Finally this value was transformed into a coefficient of variance dividing it by the mean of the distribution. This was done for each time point to generate a FB developmental sequence. The coefficient of variation approach provides a method to assess developmental timing that does not depend upon determinations of either absolute or relative fruiting body color or size.
- FB count and size. We processed the 72 h time point digital images by transforming them into a black/white binary image where each FB appeared as a black area in a white background. This transformation allows for estimation of final counts, sizes, and variation in size of FBs for each mutant. We used the coefficient of variation (CV) as a measure of heterogeneity in FB size in a developmental swarm.
- Spore count and viability. We performed total spore counts and viable spore counts by flow cytometry and plating. Fruiting bodies were harvested, by taking a plug from the agar plates containing all the FBs that developed from a single inoculum of cells. The plug was forced into 13 mm diameter tube with a sterile wood applicator and washed with 2 ml of TPM solution by vortexing. The total volume of the wash was removed using a micropipette and then sonicated to disrupt the FBs and to obtain individual spores. The sonicated spores were then incubated at 60°C for 30 min in order to kill all the non-spore cells and possible contaminants that could remain in the solution.

Total spore counts were performed on 200 µl sample from each 2 ml spore solution via a flow cytometer (Benton Dickson FACS Calibur) using a 15 mW 488 nm argon laser. Since the spores are naturally refractile no staining was needed, and the counts were obtained for 15 s. Spore count ml^−1^ (C) of the original samples was determined by:


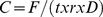
(1)

Where F is the number of spores acquired (Forward Scattered count), t is the time in seconds of data acquisition, R is the flow rate in ml*s^−1^ of the cytometer and D is the dilution performed before running the sample.

Viable spore counts were made by mixing 1 ml of spore solution with 3 ml of soft CYE agar (40°C), vortexing the mix and pouring over CYE plates. The plates were incubated for 5 days at 30°C and the resulting colonies counted as viable spores.

#### Statistical analyses

We assessed *fruA* expression by a full factorial ANCOVA (genotype, time and time^2^ as main effects), using *a priori* contrasts to compare the single knockout mutant strains versus their unmutated parental strain. Statistical significance for changes in timing of *fruA* expression was determined by a full factorial ANCOVA (mutant state, time and time^2^) on the averages of the mutant strain values and those of the unmutated parental strain.

Statistical significance for developmental traits was assessed by ANOVA, with genotype, replicate and block as main effects. All were treated as random effects, as that is statistically conservative. A replicate *X* block interaction term was included in the ANOVA, when supported by partial F-tests for improved fitting [53]. An interaction term was not automatically included due to the large reduction in degrees of freedom associated with its inclusion.

Standard mean differences (SMD) were calculated from comparisons of parental genotype (DZF1) with the knockout mutants, as described below. For each trait, the difference between the parental genotype and the average of the knockouts was determined and a 95% confidence interval calculated based upon the standard error of the values (this analysis can be done as either a t-test or an ANOVA, since there is only one degree of freedom in the numerator). The SMD estimate was calculated by dividing the trait value by the standard deviation of the unexplained error, the square root of the Mean Square Error [37]. This provides values that are in units of standard deviation, so that the results can be compared across traits. The Confidence Intervals (CIs) were computed the same way (division by the standard deviation of the explained error). In other words, the SMD are in units of phenotypic standard deviation. Confidence intervals not overlapping with zero indicate statistical differences between the parental genotype (DZF1) and the knockouts. Confidence intervals not overlapping with 1 indicates that differences between DZF1 and mutants are greater than the unexplained phenotypic variation within genotypes (Figure 3).

A principal component analysis was conducted on correlations of the six developmental traits assessed for the five genotypes. A pre-planned contrast comparing the parental genotype and the mutants was computed for each value for each PCA trait (as determined by a chi-square test), using the same ANOVA structure as was performed for the individual traits. There were three main effects, genotype, replicate and block, with genotype as a fixed effect and replicate and block considered as random factors.
